# Seroprevalence and risk factors associated with *Theileria parva* infection among calves in Narok County, Kenya

**DOI:** 10.14202/vetworld.2024.620-629

**Published:** 2024-03-17

**Authors:** Wyckliff Ngetich, George Karuoya Gitau, Tequiero Abuom Okumu, Gabriel Oluga Aboge, Daniel Muasya

**Affiliations:** 1 Department of Clinical Studies, University of Nairobi, P.O Box 29053-00625, Kangemi, Nairobi; 2 Department of Veterinary Surgery, Theriogenology and Medicine, Egerton University, P.O Box 536-20115, Egerton, Kenya; 3 Department of Public Health, Pharmacology and Toxicology, University of Nairobi, P.O Box 29053-00625, Kangemi, Nairobi

**Keywords:** calves, risk factors, seroprevalence, *Theileria parva*

## Abstract

**Background and Aim:**

East Coast fever (ECF), caused by *Theileria parva*, is a devastating disease that causes significant economic losses to cattle production in sub-Saharan Africa. Prevention and control of ECF are challenging in pastoral settings due to inadequate epidemiological information. This study aimed to estimate the seroprevalence and risk factors associated with *T. parva* infection among calves in different production systems to help design appropriate control interventions.

**Materials and Methods:**

Blood samples were collected from 318 calves and tested using an indirect enzyme-linked immunosorbent assay targeting antibodies against polymorphic immunodominant molecules found on the surface of *T. parva*. Information on calf characteristics and management practices was also collected during sampling. Descriptive statistics and logistic regression were used to analyze potential risk factors, such as age and acaricide application, where p < 0.05 was considered significant.

**Results:**

Of the 318 calves sampled, 41 (12.89%) were positive for *T. parva*, with a higher proportion in pastoral systems (36.58%) than in mixed farming systems (34.10%) and agropastoral systems (29.27%). From univariate analysis, calf age (p = 0.002), body weight (p = 0.001), suckling status (p = 0.026), rectal temperature (p = 0.06), calves on pasture (p = 0.022), other feeds (p = 0.004), feed grown within the farm (p = 0.004), acaricide application (p = 0.001), and acaricide application frequency (p = 0.001) were significantly associated with seropositivity. However, calf age (odds ratio [OR], 0.96; 95% confidence interval [CI], 0.91–0.99; p = 0.04), other feeds (OR, 8.82; 95% CI, 1.74–44.63; p = 0.009), and suckling status (OR, 0.38; 95% CI, 0.15–0.99; p = 0.05) were significantly associated with *T. parva* infection in the multivariable mixed logistic model.

**Conclusion:**

*T. parva* is circulating in young calves in the study area (and possibly in cattle populations due to maternal transfer of antibodies to the calves). There is a need for molecular surveillance to determine the presence and burden of *T. parva* infection.

## Introduction

Tick-borne diseases (TBDs) are one of the main health problems in the livestock industry, especially in cattle production systems. East Coast fever (ECF), caused by *Theileria parva*, has the most devastating effects on smallholder farmers and pastoralists [1, 2]. *Rhipicephalus appendiculatus*, whose extensive distribution in many countries in sub-Saharan Africa [3] coincides with that of ECF [4]. In the East African region, ECF has been ranked as the most important TBD, with a high mortality rate in calves less than six months [5]. ECF has been reported to be the leading cause of female calves’ mortality in Murang’a [6] and a significant cattle disease among Maasai pastoralists [1] in Kenya. ECF is a devastating disease that kills approximately 1 million cattle annually in sub-Saharan Africa, resulting in revenue losses of $596 million [7]. It drastically reduces the income returns from livestock due to reduced production, high cost of treatment and management of clinical cases, and animal deaths, which would reduce the herd size by 30% [4, 8, 9]. Climate, vector dynamics, agroecological zones (AEZs), production system, grazing management, and animal characteristics (breed, sex, and age) influence the existence and spread of *T. parva* [10]. Similarly, ECF prophylactic strategies, such as tick control or vaccination, greatly affect the transmission and establishment of *T. parva* infection in cattle [11, 12]. The indirect fluorescent antibody test and enzyme-linked immunosorbent assay (ELISA) are the most widely used diagnostic serological tests for theileriosis, and the latter has demonstrated higher sensitivity and specificity (over 95%) [4]. Indirect ELISA is commonly used where antibodies against polymorphic immunodominant molecules (PIMs) are targeted.

Most pastoralists rely almost entirely on cattle for their livelihoods, and this disease is no doubt an economic challenge that hinders the development and improvement of livestock production in affected areas [11]. Despite the various methods developed for treating and controlling ECF, it remains a major constraint to livestock improvement and a major cause of cattle mortality. This study is unique because it assesses seroprevalence in young calves less than 1 month of age, unlike other studies in older calves [11, 13, 14]. The results of this study are crucial because they act as a prerequisite for the design and implementation of effective ECF control measures.

This study aimed to estimate the seroprevalence and risk factors associated with *T. parva* infection among young calves in Narok County, Kenya.

## Materials and Methods

### Ethical approval

The study protocols and procedures were approved by the Faculty of Veterinary Medicine’s Biosafety, Animal Use and Ethics Committee (REF: FVM BAUEC/2021/316) of the University of Nairobi. Similarly, written informed consent was obtained from all the households before the start of the study.

### Study period and location

**Figure-1:**
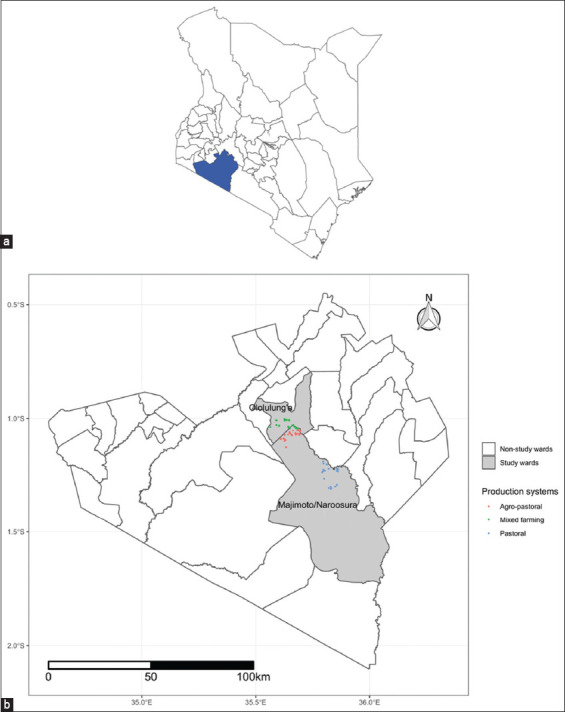
(a) Map of Kenya showing Narok County (blue). (b) The study wards with different production systems where calves were sampled. The green represents the villages in the mixed production system, the pink represents villages in agropastoral system and blue represents the villages in the pastoral production system. The map was generated from the Global Positioning System GPS coordinates captured during data collection using CommCare software (https://play.google.com/store/apps/details?id=org.commcare.dalvik&hl=en&gl=US&pli=1).

The study was conducted from December 2022 to April 2023 in Narok South Sub-County, Narok County (Figure-1a) from the two wards of Naroosura Maji Moto and Ololulung’a (Figure-1b) Two sites were selected based on high human malnutrition rates, prevalence of ECF, and different livestock production systems.

### Study design, sample collection, and processing

**Figure-2:**
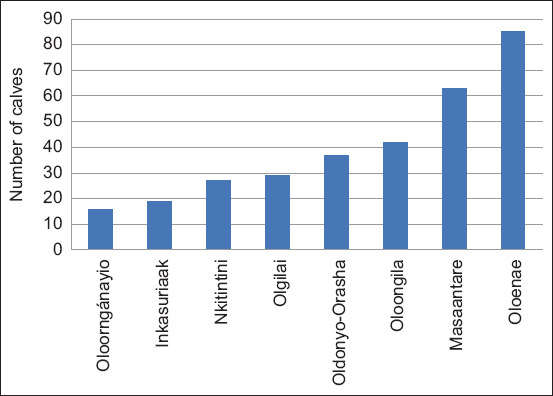
Number of calves sampled from different villages between December 2022 and April 2023 in Narok County, Kenya.

A cross-sectional design was used in this study, in which calves were randomly selected from households participating in the Feed the Future Animal Health Innovation Laboratory. A total of 318 calves were sampled from eight villages within the three different agroecosystems (Figure-2). Jugular venipuncture was used to aseptically collect approximately 8 mL of blood from each calf, labeled, and transported in a cool box containing ice packs to the field-based mini-laboratory for processing. A semi-structured questionnaire was developed, digitalized, and administered to farmers to capture information on calf characteristics, including age, sex, breed, feeding, weight, rectal temperature, and tick control practices. Similarly, tick burden was determined by identifying and enumerating the number of all visible ticks from different body parts on the selected calves based on guidelines described elsewhere [13]. The health history and treatment of the calves were recorded during sampling. Plain-tube samples were centrifuged at 1000× *g* for 15 min in a field-based mini-laboratory. The serum was transferred onto sterile cryovials and stored at –20°C awaiting analysis. Serum samples for serological analysis were submitted to the International Livestock Research Institute (ILRI, Nairobi). Indirect ELISA was used to determine *T. parva* antibody levels using a recombinant PIM, as described previously by Silatsa *et al*. [4]. For data analysis and interpretation, optical density (OD) values recorded for the test samples were expressed as a percentage of the strong positive control (C++) standard representing 100 percent positivity (PP). The computation of PP was based on OD readings from the reference positive control sera. For this study, a PP of 20 and above was considered positive for *T. parva*.

PP = OD value sample/OD C++ × 100

### Statistical analysis

Data were downloaded from the Commcare server, cleaned, and analyzed using Stata 18 software (StataCorp LLC, College Station, Texas, USA). Descriptive statistics were used to categorize antibody prevalence based on agroecosystem, calf’s characteristics such as sex, age, body weight, tick infestation status, and acaricide application and were presented in frequency tables, whereas mean, median, and range were calculated for continuous data. For the risk factors significantly associated with seropositivity outcome, p < 0.2 for univariate analysis and p < 0.05 for multivariate analysis using Fisher’s exact test was considered significant. In the first step, univariable mixed logistic models for all the predictor variables were fitted into separate generalized linear models using the functional logit. In the second step, a multivariable mixed logistic regression analysis was fitted for all the univariable associations with p < 0.20. The clustering effect was accounted for by including the herd variable in the models, and intraclass correlation coefficient was reported to be significant (residual intraclass correlation 0.216; 95% confidence interval (CI) 0.059–0.544). Correlations between predictor variables were identified using pair-wise correlation, and statistical significance and biological plausibility were used to identify the variables to remain in the final model when two variables were highly correlated (correlation coefficient >0.5). The final models were built using forward stepwise elimination, leaving those variables with p < 0.05. Explanatory variables were considered confounders if their inclusion in the final multivariable model modified the coefficients of other significant variables by 30%. The final model was evaluated for goodness of fit with and without random farm effects. The area under the receiver operating characteristic curve was 0.7379, with no evidence of lack of fit.

## Results

### Characteristics and management of the calves

More calves were sampled from the agro-pastoral system (39.94%, 127/318) compared to pastoral (31.13%, 99/318) and mixed (28.93%, 92/318) production systems Most (98.74%, 314/318) of the calves were indigenous breeds, with slightly more than half (51.57%, 164/318) of the calves being male. Most (98.74%) of the recruited calves were kept outdoors in the “bomas” at night, with most (88.05%) still suckling their mothers at the time of recruitment. Acaricides have been applied at least once in approximately 57.50% of calves, and 3.77% of calves have been reported to have a history of health problems. Most of the cattle breeds kept in the study area were indigenous, with 98.10% of the dams and 99.10% of the sires of the recruited calves being indigenous, while the rest were crossbreeds of indigenous and exotic breeds. The mean age of calves recruited was 20.70 days (median, 24 days) and ranged from 1 to 29 days. The average body weight of the calves was estimated to be 41.20 kg (range, 24–63 kg). A higher proportion (55.43%, 51/92) of the recruited calves were infested by ticks in mixed farming production systems compared to 49.61% and 23.23% in agropastoral and pure pastoral production systems, respectively. The number of acaricides applied to calves from birth to recruitment ranged from 1 to 3 times, with an average of 1.6 times. Regarding the method of acaricide application, 87.40% were sprayed on the whole body, 4.90% had a pour-on, 4.40% had an injectable, and 3.30% were sprayed on specific body parts. However, some farmers claimed that they used multiple tick control methods in 8.20% of the calves, mainly dip wash and injectables. There were statistically significant differences (p < 0.05) between variables (sick calves, presence of ticks, parasite control frequency, and average calf weight) in the different AEZs (Table-1).

**Table-1:** Characteristics and management of the 318 sampled calves by agroecosystems in Narok County.

Variable	Agropastoral (n = 127)	Mixed farming (n = 92)	Pastoral (n = 99)	Total (n = 318)	p-value
Sex (%)					0.37
Female	56 (44.09)	45 (48.91)	53 (53.54)	154 (48.43)	
Male	71 (55.91)	47 (51.09)	46 (46.46)	164 (51.57)	
Breed (%)					0.18
Cross (exotic and exotic)	0 (0.00)	1 (1.09)	0 (0.00)	1 (0.31)	
Cross (exotic and indigenous)	0 (0.00)	2 (2.17)	0 (0.00)	2 (0.63)	
Guernsey	1 (0.78)	0 (0.00)	0 (0.00)	1 (0.31)	
Indigenous	126 (99.21)	89 (96.74)	99 (100.00)	314 (98.74)	
Suckling (%)					0.67
No	13 (10.24)	11 (11.96)	14 (14.14)	38 (11.95)	
Yes	114 (89.76)	81 (88.04)	85 (85.85)	280 (88.05)	
Calf housing (%)					0.14
Indoor	1 (0.78)	0 (0.00)	3 (3.03)	4 (1.26)	
Outdoor	126 (99.21)	92 (100.0)	96 (96.97)	314 (98.74)	
Calf sick (%)					0.01
No	123 (96.85)	84 (91.30)	99 (100.0)	306 (96.23)	
Yes	4 (3.15)	8 (8.70)	0 (0.00)	12 (3.77)	
Tick infestation (%)					<0.01
Absent	64 (50.39)	41 (44.57)	76 (76.78)	181 (56.92)	
Present	63 (49.61)	51 (55.43)	23 (23.23)	137 (43.08)	
Ectoparasite control (%)					0.74
No	56 (44.09)	36 (39.13)	43 (43.43)	135 (42.45)	
Yes	71 (55.91)	56 (60.87)	56 (56.57)	183 (57.55)	
Parasite control frequency (%)					0.01
None	56 (44.09)	36 (39.13)	44 (44.44)	136 (42.77)	
Once	49 (38.58)	22 (23.91)	21 (21.21)	92 (28.93)	
Twice	20 (15.75)	28 (30.43)	26 (26.27)	74 (23.27)	
Thrice	2 (1.57)	6 (6.52)	8 (8.08)	16 (5.03)	
Weight (kg)					<0.01
Mean (SD)	41.42 (8.64)	44.39 (9.11)	38.08 (7.42)	41.24 (8.75)	
Range	26.50–63.00	28.50–62.50	24.00–62.00	24.00–63.00	
Age (days)					0.26
Mean (SD)	20.06 (7.81)	21.96 (9.19)	20.41 (9.20)	20.72 (8.67)	
Range	1.00–29.00	1.00–29.00	1.00–29.00	1.00–29.00	

p-values generated from Fisher’s exact test, SD=Standard deviation

### Feeding, watering, and housing of calves

At the time of recruitment, 50.63% (161/318) of the calves were fed solid feed, whereas the remaining calves were suckling only. For those receiving solid feed, different feeds, such as pasture (98.80%), hay (8.71%), silage (6.22%), mineral supplements (58.40%), and dairy meal (3.70%), were provided in combination. The pasture was grown on the farms, and the other feed was purchased by the farmers. With regard to access to drinking water, 58.52% of the recruited calves had access to drinking water, with 72.00% accessing drinking water from dams/cisterns within grazing areas, 9.70% within housing areas, and 18.29% from nearby streams/rivers. At night, a higher percentage (97.50%) of the recruited calves were kept in an enclosure made of untreated wood and plain/barbed wire, whereas the remaining calves were kept in bomas made of other materials such as thatch and mud.

### Health status of calves and their dams

Approximately 3.77% of calves reported a history of ill health from birth, and 83.30% of farmers reported that they knew the cause of the disease. Of those farmers who reportedly knew about the disease, 40.00% reported foot and mouth disease, 40.00% reported diarrhea, and 20.00% reported skin infection. In the case of sick calves, 75.00% received treatment either from an animal health specialist or from the farmers themselves. At the time of recruitment, 41.70% of calves with a history of disease had not fully recovered. The average age of the dams was estimated to be 5.8 years, with a median of 6.0 and a range of 2–13 years, with most dams producing approximately 1.20 L (median 1; range 0.5–6.0) of milk per day and an average parity of 2.70 (median 2; range 1–8). Approximately 4.10% of dams have been reported to have manifested ill health since giving birth, with most of them having retained the placenta.

### Tick type and distribution on the body of calves

Of all the calves, 43.08% (137/318) had ticks on their bodies, regardless of the infested body part and the type of tick present. However, no statistical significance (p = 0.056) was found for the status of calf tick infestation in different AEZs. In terms of infestation of different parts of the body, 23.89% of calves had ticks on their flanks, which is a higher proportion than infestation of other body parts. Similarly, *Boophilus* species were most common in most calves and found on all body parts. Regarding the recruitment period, a higher proportion of those recruited in March (75.36%) were infested with ticks, whereas the lowest proportion (28.73%) was reported in December (Table-2). There was a statistically significant difference (p < 0.001) between the recruitment period and tick infestation status.

**Table-2:** Number of calves infested by different types of ticks on different body parts.

Body part	Number of calves (%)	Type of tick (%)			

		Brown-ear	Blue	Red-spotted	Mixed infestation
Head					
Yes	40 (12.57)	24 (60.00)	12 (30.00)	1 (2.50)	3 (7.50)
No	278 (87.43)				
Neck					
Yes	55 (17.30)	1 (1.82)	44 (80.00)	5 (9.09)	5 (9.09)
No	263 (82.70)				
Flank					
Yes	76 (23.90)	0 (0.00)	27 (35.53)	22 (28.95)	27 (35.53)
No	242 (76.10)				
Perinium					
Yes	50 (15.72)	20 (40.00)	9 (18.00)	18 (36.00)	3 (6.00)
No	268 (84.28)				
Extremities					
Yes	63 (19.81)	2 (3.17)	36 (57.14)	14 (22.22)	11 (17.46)
No	255 (80.19)				
Calves’ tick status					

**Month**	**Number of calves sampled**	**Tick infestation (%)**			

		**Yes**		**No**	

December	87	25 (28.73)		62 (71.27)	
January	75	45 (60.00)		30 (40.00)	
February	65	45 (69.23)		20 (30.77)	
March	69	52 (75.36)		17 (24.64)	
April	22	14 (63.64)		8 (36.36)	

## Seroprevalence and description of the calves’ characteristics and management based on ELISA results

The prevalence of *T. parva* was 12.89% (41/318). However, among the three different agroecosystems, 15.22% (14/92), 15.15% (15/99), and 9.45% (12/127) of calves seroconverted from mixed farming, pastoral, and agropastoral, respectively. Of the seropositive calves, a higher proportion came from the pastoral system (36.58%; 15/41) than from the mixed farming and agropastoral systems (34.15%; 14/41) and 29.26% (12/41), respectively. More than half (58.53%) of the positive calves were male; however, most (97.56%) of the positive calves were kept outdoors in the bomas. Calf body weight was significantly associated with seropositivity (p < 0.001) because the mean weight of the positive calves was lower than that of the seronegative calves. Similarly, there was statistical significance (p = 0.002) between the age of the calf and seropositivity of *T. parva*, whereby the average age (16.80 days) of the positive calves was lower than that of the negative calves (21.30 days; Table-3). A higher proportion (75.60%) of the seropositive calves were suckling, and most (82.93%) of them did not receive acaricide treatment (Table-4).

**Table-3:** Calf characteristics and management based on ELISA results.

Variable	Negative (n = 277)	Positive (n = 41)	Total (n = 318)	p-value
Ecozone (%)				0.33
Agropastoral	115 (41.52)	12 (29.27)	127 (39.94)	
Mixed farming	78 (28.16)	14 (34.14)	92 (28.93)	
Pastoral	84 (30.32)	15 (36.59)	99 (31.13)	
Calf sex (%)				0.34
Female	137 (49.46)	17 (41.46)	154 (48.43)	
Male	140 (50.54)	24 (58.54)	164 (51.57)	
Calf housing (%)				0.47
Indoor	3 (1.08)	1 (2.44)	4 (1.26)	
Outdoor	274 (98.92)	40 (97.56)	314 (98.74)	
Calf suckling (%)				0.01
No	28 (10.11)	10 (24.39)	38 (11.95)	
Yes	249 (89.89)	31 (75.61)	280 (88.05)	
Source of drinking water (%)				0.05
During grazing/pasture	119 (42.96)	15 (36.59)	134 (42.14)	
Housing area	18 (6.50)	0 (0.00)	18 (5.66)	
Not taking water	108 (38.99)	24 (58.54)	132 (41.51)	
Stream/river	32 (11.55)	2 (4.87)	34 (10.69)	
Calf sickness (%)				0.17
No	265 (95.67)	41 (100.0)	306 (96.23)	
Yes	12 (4.33)	0 (0.00)	12 (3.77)	
Calf tick status (%)				0.57
Absent	156 (56.32)	25 (60.98)	181 (56.92)	
Present	121 (43.68)	16 (39.02)	137 (43.08)	
Acaricide application (%)				<0.01
No	101 (36.46)	34 (82.93)	135 (42.45)	
Yes	176 (63.54)	7 (17.07)	183 (57.55)	
Acaricide application frequency (%)				<0.01
None	102 (36.82)	34 (82.92)	136 (42.77)	
Once	89 (32.13)	3 (7.32)	92 (28.93)	
Thrice	15 (5.42)	1 (2.44)	16 (5.03)	
Twice	71 (25.63)	3 (7.32)	74 (23.27)	
Calf weight				<0.01
Mean (SD)	41.87 (8.90)	36.96 (6.21)	41.24 (8.75)	
Range	24.00–63.00	26.50–59.00	24.00–63.00	
Calf age				<0.01
Mean (SD)	21.30 (8.53)	16.81 (8.73)	20.72 (8.68)	
Range	1.00–29.00	2.00–29.00	1.00–29.00	

p-values generated from Fisher’s exact test, ELISA=Enzyme-linked immunosorbent assay, SD=Standard deviation

**Table-4:** Comparison of *Theilera parva* seropositivity of calves from different agro-ecosystems by parasite control frequency

Frequency of tick control	Mixed farming (n = 92)		Agro-pastoral (n = 127)		Pure Pastoral (n = 99)		Total calves (%)
		
	Elisa+ve	Elisa -ve	Elisa+ve	Elisa -ve	Elisa+ve	Elisa -ve	
Frequency of tick control							
None	12	24	12	44	10	34	136 (42.77)
Once	0	22	0	49	3	18	92 (28.93)
Twice	2	26	0	20	1	25	74 (23.27)
Thrice	0	6	0	2	1	7	16 (5.03)
Total	14	78	12	115	15	84	318 (100)

ELISA +ve means samples with >20 percent positive (PP) (seropositive) while ELISA -ve are samples with <20 PP (seronegative)

### Factors associated with *T. parva* seroprevalence

Variables related to calf characteristics and management were collected and analyzed to identify various risk factors. Table-5 summarizes the variables that were found to be statistically associated with seropositivity to *T. parva* infection at p < 0.2.

**Table-5:** Results of univariable logistic regression analysis.

Variable	OR	95% CI	p-value
Calf age >21 days	0.94	0.90–0.98	<0.01
Calf age <21 days	Ref		
Suckling status Yes	0.35	0.14–0.88	0.03
Suckling status No	Ref		
Exclusive suckling Yes	2.50	1.18–5.68	0.02
Exclusive suckling No	Ref		
Calves on pasture Yes	0.40	0.18–0.88	0.02
Calves on pasture No	Ref		
Mineral supplementation Yes	0.15	0.04–0.55	<0.01
Mineral supplementation No	Ref		
Providing other feeds Yes	9.10	0.19–3.77	<0.01
Providing other feeds No	Ref		
Feed grown in the farm Yes	0.32	0.15–0.69	<0.01
Feed grown in the farm No	Ref		
Acaricide application Yes	0.12	0.04–0.28	<0.01
Acaricide application No	Ref		
Rectal temperature >39.5°C	1.77	0.98–3.20	0.06
Rectal temperature <39.5°C	Ref		
Calf weight >41 kg	0.92	0.87–0.97	<0.01
Calf weight <41 kg	Ref		

OR=Odds ratio, CI=Confidence interval

The level of significance during the multilevel mixed effect logistic regression model analysis was set at p < 0.05. This analysis yielded three significant factors associated with the risk of *T. parva* infection: age of the calf (odds ratio [OR] 0.96; 95% CI 0.91–0.99, p = 0.04), suckling status (OR 0.38; 95% CI 0.15–0.99, p = 0.05), and providing other feeds apart from pasture (OR 8.82; 95% CI 1.74–44.63, p = 0.009). Among these factors, the age of the calves was categorized as <21 days and more than 21 days. In comparison with younger calves, older calves (more than 21 days) had a 0.96 probability of testing positive. Similarly, suckling was protective as calves that were suckling had a 62% reduction in the probability of testing positive. On the other hand, feeding calves other feeds (apart from pasture) had 8.80 odds of testing positive. The other variables did not significantly predict ELISA results for *T. parva* in calves.

## Discussion

This study was conducted to determine the presence of *T. parva* antibodies in young calves in Narok. We also investigated potential risk factors associated with *T. parva* infection as a crucial prerequisite for designing and implementing effective ECF control measures. The presence of antibody titers at 20% and above is an indication of exposure and may not indicate an active infection (seroconversion), as calves may have passively transferred antibodies (colostrum) from previously infected dams [15]. Longitudinal seroconversion studies have shown that calves may be infected within the first few weeks of life [16]. However, the prevalence reported in this study is lower than that reported elsewhere. In a cross-sectional serological study conducted in Machakos, *T. parva* antibodies were detected in 40.90% of the sampled animals [14]. However, unlike in the current study, their study sampled animals older than 4 months. In Maasai communities living in Tanzania, Kimaro *et al*. [17] reported 100% seroprevalence to *T. parva* infection in cattle older than 3 months. This suggests a high overall prevalence and presumably endemic stability in pastoral cattle herds, even if some cattle are vaccinated. In another study conducted in Uganda to determine the prevalence of *T. parva* infection in calves interacting with vaccinated cattle, 27.40% were positive, suggesting a possible carrier status and cross-infection [5]. However, the prevalence reported in this study is higher than the 5.0% reported in Tanzania [11]. This difference could be attributed to the polymerase chain reaction test used on the samples, which detects the presence of genetic material for *T. parva*. Similarly, a few calves with a history of ill health were sampled, unlike in Tanzania, where the cattle sampled were described as healthy at the time of sampling.

In this study, older calves (more than 21 days old) were less likely to test positive compared with younger calves. In principle, it agrees with what has been reported in Northern Tanzania that younger cattle (<24 months old) are 3 times more likely to test positive than older cattle [17]; however, the cutoff age is different. However, these findings differ from those of other studies[18, 19]. In a study on calves grazing within a protected area in Uganda, the prevalence of *T. parva* in calves aged <3 months (45%) was significantly lower than that in calves aged more than 3 months (92%) [18]. Similarly, in Pakistan, adult cattle older than 2 years were found to have a higher likelihood of testing positive for *T. parva* infection than younger cattle [19]. This difference can be attributed to the influence of maternal antibodies in this study; however, other studies have shown that adult animals are more likely to have been exposed to infected ticks during grazing and have a weak immune response [20] than young cattle [21]. Unlike other important tick-borne pathogens, such as *Babesia* and *Anaplasma*, which cause severe disease in adult cattle but mild clinical signs in calves, allowing for the establishment of endemic stability in the affected regions [22], there is no sufficient evidence of age-associated resistance to *T. parva* infection. However, cattle that recover from parasite infection in the field develop immunity that can be improved by further parasite exposure.

A higher proportion of calves that tested positive were from the pastoral production system. In pastoral settings, there is no demarcation of the grazing areas, and herds from different households/villages graze together in communal grazing fields and protected wildlife areas, which increases the exposure to *T. parva* infection. In addition, there are increased livestock movements in pastoral settings in search of pasture and water where cattle tend to pick infected ticks, increasing the risk of infection [23]. To a certain extent, these findings agree with the previous study by Wesonga *et al*. [14] of substantial variability in serum antibody prevalence from different grazing systems and AEZs. Cattle from semi-arid to arid areas had a higher risk of infection than those from semi-humid areas because farmers in drier areas reared animals under a free grazing system with the potential interaction of different herds [1, 14]. Similarly, a review of Tanzanian studies reported a higher prevalence of ECF (27.70%–77.50%) in agropastoral systems compared to 2.70% in mixed/zero-grazing production systems [24]. On the other hand, other studies reported a higher prevalence (50.40%) of ECF in pastoral areas of Maasai community citing insufficient tick control measures and proximity to protected wildlife areas as the main drivers [25]. However, other studies using molecular detection have reported a higher prevalence, ranging from 7.40% to 60.10% [5, 13, 23, 24].

The suckling status of the calf was statistically significant according to the ELISA results, which can be attributed to the possible transfer of antibodies from the dam through the colostrum, thereby increasing the antibody titer [15, 16]. This may also indicate that *T. parva* is circulating in the cattle population and that the dams have previously been exposed to *T. parva* and have developed immunity. However, the passive transfer of antibodies from dams to calves is not protective because ECF immunity is cell-mediated and the calves are still susceptible to infection.

Approximately 43% of the calves in the present study were infested with ticks, which is higher than that reported in cattle in the livestock-wildlife interface of the Serengeti National Reserve [11]. However, it is <92% reported in northern Tanzania [23] and 50% in Mara [13]. Although ECF is a TBD, the tick infestation status of the calves was not statistically significant. This may be attributed to the young calves sampled in this study, and seropositivity may be caused by maternal antibodies. Similarly, *R. appendiculatus* is a three-host tick and spends only a few days on one animal before it drops off and changes into the next stage of development and we may have missed the ticks. However, it has been reported by Wesonga *et al*. [14] that cattle infested with *R. appendiculatus* have a higher risk of exposure than those not infested. *R. appendiculatus* was found, apart from the ear and perineum, in the neck and extremities, which are normally not historical predilection sites [23]. However, the type and frequency of acaricide application also significantly affects tick infestation status. In this respect, acaricide application was statistically associated with the prevalence of *T. parva*, as calves sprayed were less likely to test positive than those not sprayed. This is in line with what has been reported in Tanzania [11], that regular tick control with acaricides significantly reduces the burden on selected animals and the prevalence of ECF. In contrast, [1] claimed that farmers’ perceptions of ECF risks and risk aversion, which are mainly based on previous experience, can motivate the implementation of tick management techniques on farms, regardless of the burden of ticks. Although acaricide application and frequency were associated with lower odds of seropositivity, they were not included in the final model, suggesting that acaricides may not have a significant protective effect and the possibility of maternal antibody influence, in which acaricides have no direct effect. Similar findings have been reported elsewhere that acaricide application may indicate that incorrect spraying (acaricide misuse, inappropriate dilution, counterfeit products, and potential tick resistance) is an ineffective tick deterrent practice [13].

Most (98.74%) of calves were indigenous breeds kept outdoors at night. Similar observations have been reported from studies in the Tana River and Narok counties of Kenya, where cattle breeds were predominantly indigenous [26]. In addition [27], pastoral communities in Kenya preferred local (indigenous) cattle breeds due to their resilience and reproductive ability. The baseline survey for this study also revealed that 86.80% of the participating households kept indigenous cattle breeds [28]. In Tanzania [24], Ethiopia [29], and Botswana [30], pastoral communities living in arid and semi-arid lands preferred indigenous cattle breeds as appropriate breeds. In these areas, rainfall patterns are not predictable, and in the recent past, there has been a prolonged drought, which has necessitated a large number of animal movements in search of pasture and water. Exotic cattle breeds have been reported to be more susceptible to ECF than indigenous breeds [19] that are regularly exposed to ticks and tick-borne pathogens that contribute to protective immunity against TBD [31]. However, the breed was not statistically associated with seropositivity, which can be attributed to a large discrepancy because only 1.25% of the calves were crossbreeds and, therefore, not representative.

In the present study, 50.63% of the calves were fed solid feed, mainly pasture (98.80%) grown within the farm, and a few received supplementary feeding such as silage, dairy meal, and minerals. Similar findings have been reported elsewhere that natural pasture is the main feed resource for cattle [32–34]. The type and source of feed were significantly associated with seropositivity because calves fed on pasture grown on the farm were less likely to test positive compared to calves fed on other feed or taken for grazing purposes. Calves taken for grazing are likely to come into contact with other herds or infected ticks and, therefore, have a greater risk of infection. This is in line with the findings of other studies that animals on free grazing have a higher risk of ECF than stall feeding [19]. If feed is obtained from other farms, infected ticks may also be introduced, which will act as a source of infection for calves.

## Conclusion

Our results suggest that *T. parva* is circulating in cattle populations and that young calves have been actively or passively exposed to *T. parva*. Unexpectedly, the seroprevalence of *T. parva* is almost similar in pastoral and mixed farming systems and lower in agropastoral systems. Risk factors, such as calf age (young), not suckling, and feeding on other feeds, significantly enhanced the chances of *T. parva* infection. It is recommended that molecular analysis should be performed to accurately detect and identify *T. parva* parasites and determine their true prevalence. Similarly, calves should be followed and serially sampled to determine seroconversion and estimate the incidence rate of both ECF and *T. parva* infection. The genetic profile/diversity of circulating *T. parva* in the cattle population needs to be determined.

## Authors’ Contributions

WN, GKG, TAO, and GOA: Conceptualization and methodology, WN: Data collection and drafted the manuscript. WN and DM: Data analysis, GKG, TAO, GOA, and DM: Review and editing of the manuscript. All authors have read, reviewed, and approved the final manuscript.
